# Metabolic Interactions between Vitamin A and Conjugated Linoleic Acid

**DOI:** 10.3390/nu6031262

**Published:** 2014-03-24

**Authors:** Gianfranca Carta, Elisabetta Murru, Lina Cordeddu, Berenice Ortiz, Elena Giordano, Martha A. Belury, Loredana Quadro, Sebastiano Banni

**Affiliations:** 1 Department of Biomedical Sciences, University of Cagliari, Cittadella Universitaria, S.S. 554, km. 4500, Monserrato, Cagliari 09042, Italy; E-Mails: giacarta28@hotmail.com (G.C.); m.elisabetta.murru@gmail.com (E.M.); 2 Department of Bioscience and Nutrition, Karolinska Institute, Stockholm 141 83, Sweden; E-Mail: lina_c@hotmail.it; 3 Gerstner Sloan-Kettering Graduate School in Biomedical Sciences, Memorial Sloan-Kettering Cancer Center, New York, NY 10065, USA; E-Mail: OrtizB@mskcc.org; 4 Laboratory of Functional Foods, Madrid Institute for Advanced Studies (IMDEA)-Food, CEI UAM + CSIC, Madrid 28049, Spain; E-Mail: elen79@yahoo.it; 5 Department of Human Nutrition, Ohio State University, Columbus, OH 43210, USA; E-Mail: belury.1@osu.edu; 6 Department of Food Science and Rutgers Center for Lipid Research, Rutgers University, New Brunswick, NJ 08901, USA; E-Mail: quadro@AESOP.Rutgers.edu

**Keywords:** conjugated linoleic acid (CLA), retinol, vitamin A, retinol binding protein (RBP), oleoylethanolamide (OEA), palmitoylethanolamide (PEA), peroxisome proliferator-activated receptor-alpha (PPARα)

## Abstract

Lipid-soluble molecules share several aspects of their physiology due to their common adaptations to a hydrophilic environment, and may interact to regulate their action in a tissue-specific manner. Dietary conjugated linoleic acid (CLA) is a fatty acid with a conjugated diene structure that is found in low concentrations in ruminant products and available as a nutritional supplement. CLA has been shown to increase tissue levels of retinol (vitamin A alcohol) and its sole specific circulating carrier protein retinol-binding protein (RBP or RBP4). However, the precise mechanism of this action has not been elucidated yet. Here, we provide a summary of the current knowledge in this specific area of research and speculate that retinol and CLA may compete for catabolic pathways modulated by the activity of PPAR-α and RXR heterodimer. We also present preliminary data that may position PPAR-α at the crossroads between the metabolism of lipids and vitamin A.

## 1. Introduction

Conjugated linoleic acid (CLA) is a group of unusual fatty acids containing conjugated double bonds. The CLA present in mammalian tissues is derived from the diet or, in a smaller amount, from the intestinal flora [1]. CLA is produced by bacteria within the rumen or upon δ-9 desaturation of vaccenic acid, another fatty acid produced by ruminal bacteria [2]. Therefore, the main dietary sources of CLA are dairy products and the meat of ruminants [3].

The predominant “natural” isomer of CLA, *cis* 9, *trans* 11 (CLA c9, t11), exists in food among a plethora of other isomers that are present in much lower concentrations. After the discovery that CLA possesses anticarcinogenic activity [4], the need to produce large amounts of CLA prompted the production of synthetic CLA by alkali isomerization of linoleic acid [5]. This process resulted in the formation of several isomers, among which the CLA c9, t11 and the CLA t10, c12 were the most abundant. With such CLA preparations, several studies were carried out to investigate its anti-carcinogenic, anti-atherogenic, and anti-adiposity biological activities [6]. Some of the biological properties of CLA are specifically attributed to the t10, c12 isomer. For instance, CLA t10, c12 has the unique capability to inhibit lipid accumulation in adipose tissue, likely through downregulation of the peroxisome proliferator-activated receptor-γ (PPAR-γ) via activation of upstream signals which may induce integrated stress response (ISR), free fatty acid release and activation of NFκB and MAPKs that may directly antagonize PPAR-γ activity [7]. In addition, it has been shown that activation of AMPK by t10, c12 CLA may also be involved in adipocyte lipid reduction [8]. In contrast, CLA properties linked to the activation of PPAR-α are equally shared by the c9, t11 and t10, c12 CLA isomers [9], and it seems not directly correlated to the antiadiposity activity exerted by t10, c12 CLA [10].

PPARs are ligand-activated nuclear receptors involved in the transcriptional regulation of lipid and energy homeostasis [11]. Three isoforms are known with a high degree of sequence conservation across various species: PPAR-α, PPAR-β/δ and PPAR-γ [11]. In humans and mice, the three PPAR isoforms are encoded by separate genes on different chromosomes [12]. PPARs function as sensors for fatty acids and fatty acid derivatives, and control metabolic pathways involved in energy homeostasis [13]. The three PPAR isoforms exhibit distinct and non-interchangeable functional roles in energy metabolism [12]. PPAR-α predominantly regulates lipid catabolism. In the liver, PPAR-α regulates mitochondrial, peroxisomal, and microsomal fatty acid oxidation, as well as senses the influx of fatty acids during fasting to enhance the fatty acid burning capacity [14]. Activation of PPAR-β/δ also induces expression of genes required for fatty acid oxidation and energy dissipation in skeletal muscle and adipose tissue, which in turn lead to improved lipid profiles and reduced adiposity [15]. In the liver, PPAR-β/δ can be activated by plasma free fatty acids influxed during fasting [16]. PPAR-γ is an essential regulator of adipogenesis and fat storage in adipocytes [17].

Not all fatty acids have similar binding affinity for PPARs [18]. Therefore, modulation of PPAR activity is dependent on the intracellular total fatty acid concentrations as well as their specific profile. CLA is a strong ligand of PPARα [9] and is believed to exert some of its metabolic effects by activating this receptor [6]. In competitive binding scintillation proximity assays, CLA isomers bind to PPARα with submicromolar IC_50_ values (140 nM to 400 nM) [9]. Although one cannot directly compare the IC_50_s obtained in different studies, due to different methodologies and/or receptor constructs utilized, CLA isomers appear to have an IC_50_ similar to that of the most avid fatty acid ligands of PPARα reported, such as that of oxygenated eicosanoids [19]. Interestingly, most of the naturally occurring fatty acids have lower affinity for PPARα (IC_50_ ~1 μM [20]).

In an attempt to evaluate the link between CLA metabolism and mammary anticarcinogenic activity, we serendipitously discovered that tissue levels of retinol, vitamin A alcohol derivative, were increased in rats fed a mixture of CLA isomers (primarily c9, t11 and t10, c12) [21]. This prompted us to investigate the possible mechanism(s) of action of enhanced retinol levels and to determine whether the altered vitamin A levels are physiologically significant.

## 2. Vitamin A Metabolism and Functions

Vitamin A is an essential nutrient that controls many crucial biological functions such as vision, reproduction, development, growth, and immunity [22,23]. Vitamin A exerts its functions through oxidized metabolites of retinol: retinaldehyde and retinoic acid. Retinaldehyde is involved in the visual cycle [24], and retinoic acid regulates the expression of many target genes through receptor-mediated events [25,26,27,28,29,30]. In animals, vitamin A and its derivatives are derived from animal products as preformed dietary vitamin A (retinyl esters, retinol and very small amounts of retinoic acid), or as β-carotene from vegetables and fruits [31].

Within the intestinal mucosa all retinol, regardless of its dietary origin, is enzymatically re-esterified with long-chain fatty acids. Together with other dietary lipids, the newly synthesized retinyl esters are packaged into chylomicrons and secreted into the lymphatic system [32]. Once in the general circulation, nascent chylomicrons undergo lipolysis of the triglycerides giving rise to free fatty acids and chylomicron remnants [33]. Approximately 75% of chylomicron-retinoids are cleared by the liver, the major site of vitamin A storage and metabolism [34,35]. The remaining 25% are cleared by extrahepatic tissues [36].

To meet tissue vitamin A needs the liver secretes retinol into the circulation bound to its specific transport protein, retinol-binding protein (RBP or RBP4) [37,38]. RBP is a 21 kDa protein with a single binding site for one molecule of all-*trans*-retinol. The major function of RBP is to mobilize hepatic vitamin A stores and deliver retinol to peripheral tissues [37,39]. It is mainly, but not exclusively, synthesized within hepatocytes [37,38]. RBP circulates in the blood as a 1:1 molar complex with another serum protein, transthyretin (TTR) [40]. In the fasting circulation, retinol-RBP accounts for approximately 99% of all serum retinoids. In the postprandial circulation, the concentration of chylomicrons and chylomicron remnants with retinoids can greatly exceed the concentration of plasma retinol. Blood levels of retinol-RBP in both humans and animals are maintained very constant, except in extreme cases of nutrition and in certain disease states [22,41].

## 3. Influence of Dietary CLA on Retinol and RBP Tissue Levels

In the first report on the effects of dietary CLA on mammalian tissue, we found increased retinol levels in plasma, mammary tissue and liver of female rats fed increasing concentration of CLA (0%–2%) [21]. More specifically both retinol and retinyl ester levels were elevated upon CLA feeding, with only liver retinol levels increasing in a CLA dose-dependent manner [21]. In order to meet tissue retinoid needs, the liver secretes retinol-RBP into the bloodstream [37]. We first became interested in the influence of CLA on RBP when a report demonstrated an increase of RBP mRNA in pig liver upon CLA feeding [42]. We therefore investigated whether the peculiar effect of dietary CLA on retinol metabolism was RBP-mediated. Additionally, in contrast to the majority of the literature data obtained by feeding laboratory animals with an equimolar mixture of CLA c9, t11 and CLA t10, c12, we sought to establish whether CLA c9, t11 and CLA t10, c12 had distinct and/or common effects on vitamin A metabolism.

We performed our study using wild-type and RBP knockout (RBP KO) mice chronically fed with separate enriched preparations of the two CLA isomers [43]. We showed that consumption of CLA c9, t11 or CLA t10, c12 induced hepatic retinyl ester accumulation in wild-type mice [43]. Only the CLA t10, c12 isomer increased hepatic retinol secretion and elevated the levels of serum retinol and its carrier RBP. In addition, we demonstrated that chronic intake of CLA t10, c12 redistributed retinoids from the hepatic stores toward the adipose tissue via RBP [43]. RBP KO mice did not exhibit increased serum retinol levels or redistribution of hepatic stores when fed the CLA t10, c12, confirming the crucial role of RBP in mediating the changes of vitamin A metabolism [43]. Within this study, we also provided evidence that dietary CLA t10, c12 could stimulate hepatic retinoid oxidation, as indicated by the upregulation of the mRNA levels of Cyp26A1 and Cyp2c39 in the liver of the wild-type mice. These two enzymes are members of the cytochrome P450 (CYP) family of enzymes and contribute to retinoid hemostasis by oxidizing retinoic acid into more polar inactive metabolites [44,45]. It is interesting to note that the expression of CYP isoforms is regulated by PPAR-α [46] of which CLA isomers are strong ligands [9]. Specifically, CLA has been shown to increase CYP4A1 expression in liver [47]. Furthermore, the chronic feeding of mice (and rats) with CLA resulted in a dramatic reduction of adipose tissue and a concomitant massive fat deposition in the liver [48]. Based on this evidence, we speculated that the stimulatory effect of dietary CLA t10, c12 on hepatic retinol secretion and probably oxidation of retinoids may be part of a compensatory mechanism of the tissue to counter the excessive accumulation of lipids, including retinoids. Remarkably, serum RBP levels have been shown to be elevated in patients with nonalcoholic fatty liver disease [49].

Short-term intake of CLA also alters vitamin A metabolism. We demonstrated this by administering a bolus dose of radiolabeled retinol in combination with either CLA c9, t11 or CLA t10, c12 to both WT and RBP KO mice [50]. Upon ingestion of either CLA isomer hepatic accumulation of retinoids took place rapidly (only three hours after gavage), and recently ingested vitamin A was rapidly re-secreted from the liver mainly in the form of retinol likely bound to RBP [50]. In addition, the use of an inhibitor of the clearance of chylomicrons suggested that CLA intake rapidly enhances intestinal absorption of dietary vitamin A, and that the vitamin A that accumulates in the liver upon CLA intake may be of intestinal origin [50].

Overall, these studies showed a dramatic effect of dietary CLA on murine retinoid metabolism. However, the molecular details of such interference await a study that would full unravel them.

How does CLA regulate the secretion of the complex retinol-RBP? How does CLA seem to promote hepatic retinoid oxidation and intestinal vitamin A absorption? These and other questions need to be answered in order to understand the real biological implications of this nutrient-nutrient interaction. In the paragraph below we speculate on how some of these interactions may take place.

## 4. Is PPAR-α the Key Regulator of CLA and Retinoid Metabolic Routes?

Lipid-soluble molecules share several aspects of their physiology due to common adaptations that allow them to function in a hydrophilic environment. Absorption, transport, tissue storage, receptor-binding activities, metabolism and catabolism need specific strategies to overcome lipophilicity and differences in polarity. Molecules that share these strategies include dietary vitamin A and fatty acids. Their biological functions are exerted through binding to specific nuclear receptors. Interestingly, the fatty acid receptor [51] PPAR-α and the retinoid X receptor, RXR, must form heterodimers to regulate gene expression, implying that their action is strictly regulated by the intracellular concentration of both fatty acids and retinoids. While it is clear that fatty acids are the ligands for PPARα [52], it is still debated which are the endogenous ligands of RXR [53]. The 9-*cis* isomer of retinoic acid was initially considered the endogenous ligand of RXR, but the inability to detect this compound *in vivo* in all the tissues has raised some concerns. Interestingly, it has been demonstrated that several fatty acids can also bind this nuclear receptor at low microM [54], which may enhance the transcriptional activity of RXR-PPARs heterodimer.

RXR activation has been shown to enhance catabolic pathways regulated by PPAR-α, including the catabolism of lipid molecules such as eicosanoids via peroxisomal β-oxidation, and xenobiotics, retinoids and fatty acids by the activity of the CYP enzymes [51,55]. It is known that catabolism of CLA is predominantly mediated by peroxisomal β-oxidation, while retinoids are preferentially catabolized via the action of the CYP enzymes [45]. However, there is solid evidence indicating that different lipid molecules compete for these same two pathways. For example, it has been clearly shown that CLA and isoprostanes compete for peroxisomal β-oxidation, [56] and competition for CYP action has been well demonstrated between several drugs and alcohol [57]. We hypothesize that a competition for common catabolic pathways activated by PPAR-α may explain, at least in part, the interference between dietary CLA and tissue retinoid metabolism. Preliminary data from our laboratories seem to support this possibility. Feeding wild-type mice with WY-14,643 (0.01%), a potent synthetic PPAR-α inducer [58], dramatically decreased liver retinol and retinyl esters content (Table 1), possibly due to an increased PPAR-α-mediated CYP activity, even though we cannot rule out that the decrease may be related to other causes, such as decreased dietary uptake and/or increased mobilization to extrahepatic tissues. While a decrease of retinyl esters may be ascribed to a possible increase in retinyl ester hydrolase activity. In contrast, when mice were fed a 1:1 mixture of CLA c9, t11 and CLA t10, c12 (1.5%), known endogenous PPAR-α ligands, hepatic retinoid levels increased (Table 1), as previously shown [21]. This discrepancy could be explained by a competition for the same catabolic pathway that takes place between retinoids and CLA, but not WY-14,643. Even though it has been shown that fatty acids are metabolized by the CYP enzymes [59], to our knowledge there is no data on CLA as substrate for these enzymes. Therefore, the competition for CYP enzymes proposed above is only a potential possibility and further studies should be carried out to prove that it actually takes place. We speculate that if CLA undergoes degradation by CYP action, which is likely stimulated by CLA through PPAR-α activation [47], hepatic retinoids would indeed accumulate. To our knowledge, it is not known how WY-14,643 is metabolized within the body. However, given the extremely low concentration of this drug in the diet fed to our mice, we speculate that it is unlikely that WY-14,643 could compete with retinoids for degradation by CYP activity

**Table 1:** Retinol and retinyl esters concentration in liver of female mice fed for 42 weeks control diet (CTRL), or CTRL diet + 1.5% of conjugated linoleic acid (CLA) (mixture 1:1 of c9, t11 and t10, c12), or CTRL diet + 0.01% of WY-14,643.

Treatment	Retinol	Retinyl Esters
	μg/g of Liver	
CTRL	4.69 ± 0.73 ^a^	273.69 ± 67.37 ^a^
CLA	6.68 ± 2.03 ^a^	470.64 ± 46.06 ^b^
WY-14,643	1.41 ± 0.45 ^b^	75.74 ± 14.23 ^c^

^a,b,c^ Different letters denote significant differences (*p* < 0.05) with one-way ANOVA and Tukey post-hoc analysis.

It is interesting that PPAR-α could directly regulate RBP expression, at least in certain tissues [60]. This data suggests that the ability of CLA to increase the levels of hepatic and circulating RBP could be the result of a direct action of PPAR-α on RBP levels. On the other hand, in a sort of positive feedback mechanism, the increase of RBP levels may potentially enhance retinoid availability for RXR-PPARα action, by promoting retinol transport to the periphery of the body. In this sense, RBP could be essential to amplify the PPAR-α-mediated metabolic pathways, including those involved in energy homeostasis. We have evidence that RBP could indeed play such an important role.

**Figure 1:**
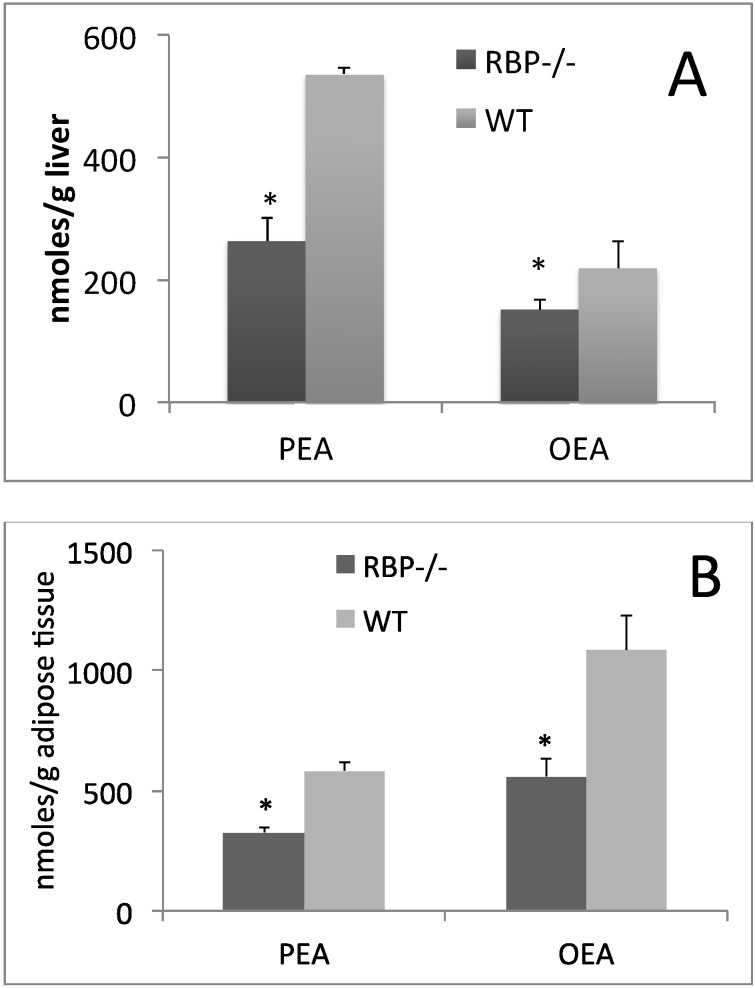
Palmitoylethanolamide (PEA) and oleoylethanolamide (OEA) levels in liver (**A**) and adipose tissue (**B**) of RBP^−/−^ or wild type mice. * significant difference (*p* < 0.05) with *t*-student test.

We have recently shown that PPAR-α activation in rodent brain slices increases the biosynthesis of the endocannabinoid congeners, oleoylethanolamide (OEA) and palmitoylethanolamide (PEA) [61]. Interestingly, both OEA and PEA also induce PPAR-α affecting energy homeostasis [62,63], probably by sustaining PPARα activity with a positive feedback. Also, PEA is a TRPV1 ligand [64]. Our preliminary data show that RBP KO mice have lower levels of OEA and PEA in the liver (Figure 1A) and adipose tissue (Figure 1B) when compared to age- and sex-matched wild-type animals. Whether CLA feeding, through induction PPAR-α activity and thus RBP expression, also enhances PEA and OEA tissue levels is not known at the moment and it would be an important question to address.

## 5. Conclusions

The influence of dietary CLA on retinol metabolism is yet another example of nutrient-nutrient interaction that points to the difficulties in isolating and/or claiming the nutritional property of a single dietary component, without taking into account all the possible interactions with other nutrients. Once all the molecular mechanisms of the interaction between CLA and retinol are elucidated, novel dietary strategies can be explored to regulate retinol and fatty acid metabolism by modulating the heterodimer PPAR-α-RXR activities in different tissues to promote health in humans.
